# Effect of *CSN3* Gene Polymorphism on the Formation of Milk Gels Induced by Physical, Chemical, and Biotechnological Factors

**DOI:** 10.3390/foods12091767

**Published:** 2023-04-24

**Authors:** Aleksandr G. Kruchinin, Elena E. Illarionova, Aram G. Galstyan, Svetlana N. Turovskaya, Alana V. Bigaeva, Ekaterina I. Bolshakova, Mariya N. Strizhko

**Affiliations:** All-Russian Dairy Research Institute, Lusinovskaya Str. 35 (Blok 7), 115093 Moscow, Russia

**Keywords:** milk, *CSN3*, polymorphism, concentration, enzymes, coagulation

## Abstract

During the last decade, research into genetic markers in the casein gene cluster has been actively introduced in cattle breeding programs. A special interest has been paid to the polymorphism of the *CSN3* gene, responsible for the expression of the k-casein, playing a key role in protein coagulation, interaction with whey proteins, stabilization, and aggregation of casein micelles. This paper aimed to determine the effect of *CSN3* genetic polymorphism on acid; rennet; acid–rennet; heat- and acid-induced as well as heat- and calcium-induced coagulation in skimmed milk; and protein-standardized milk systems (UF, NF, RO, VE). The influence of polymorphic variants of the *CSN3* gene on the coagulation ability of milk proteins was assessed by the particle size of casein micelles, protein retention factor in the clot, and coagulation ability (duration of induction period, mass coagulation period, dynamic viscosity in gel point). The correlation between *CSN3* gene polymorphism and protein coagulation was revealed. Milk systems obtained from *CSN3 BB* milk were found to have the shortest duration of coagulation, formation of better gel strength values, and increased yield compared to *CSN3 AA*. This study will improve the efficiency of milk processing and optimize the technology of dairy product production.

## 1. Introduction

Cheese is one of the most consumed dairy products in the world. It is predicted that in the five-year outlook, the annual increase in consumption of cheese will average 1.4%. Currently, the world’s production of cheese is more than 19 million tons per year (35–40% of all processed milk), and has about 3000 cheese types [1,2]. A wide range of cheeses is caused by a variety of technological, biochemical, and microbiological processes that form individual identification features of the final product [3,4]. With the growth of intensification processes in cheese making, the requirements for the sustainability of quality, maintaining identification properties of final products, and efficiency of milk raw material processing have become stricter. This causes the demand for scientific research aimed at a comprehensive study of coagulation and gel strength [5].

To date, it is generally acknowledged that the efficiency of cheese production is influenced by many interrelated factors. These include genotypic and phenotypic characteristics of animals, paratypic features (feeding ration, housing period and conditions, lactation period, age of the animal, etc.), the physical and chemical composition of milk, the ratio of casein and whey proteins, distribution of milk salts (Ca, Mg, and P) between micellar and whey phases, coagulation method, used rennet, starter cultures species, technological and biochemical processes during cheese production, and ripening [4,6,7,8,9]. During the last decade, research into genetic markers in the casein gene cluster has been actively introduced in the programs of selective breeding of cattle. The reason is that these markers are associated with milk productivity, fat content, certain fractions of milk protein and minerals, heat stability, coagulation ability of milk, and gel strength [10]. Caseins (αS1-, αS2-, β-, and k-casein) account for about 80% of the protein content in milk and are encoded by four related genes *CSN1S1*, *CSN1S2*, *CSN2*, and *CSN3*, united in a cluster of about 250 Kb, which in cattle is located in chromosome 6. A high degree of polymorphism in each of the four genes has a different effect on cow productivity and milk quality. At present, the polymorphism of the *CSN3* gene has been intensively studied, due to its responsibility for the k-casein expression, with a special location in the structure of casein micelles and its technological functionality [11,12,13]. Casein micelles are colloidal particles of 50 to 500 nm in diameter with an average mass of 10^6^ to 10^9^ Da [14]. Almost half of the micelle surface layer is occupied by k-casein (about 45%). Its hydrophilic C-terminal part, 5–10 nm long, provides the micelle with steric stabilization in solution due to its zeta potential (about −20 mV at pH 6.7) [14,15,16]. The k-casein molecule, consisting of 169 amino acids, contains a site (between 105 and 106 amino acids) specific for hydrolysis by chymosin (rennet enzyme). Hydrolysis of about 80% of k-casein causes loss of steric protection of micelles and initiates a process of aggregation in the presence of calcium ions. This results in the formation of a paracasein matrix, which includes fat globules and part of the soluble phase of milk [17]. Thus, k-casein plays a key role in cheese production, influencing the yield and characteristics of the clot, while its genetic polymorphism may have a significant impact on the technological process [13].

Currently, 14 polymorphic variants of the *CSN3* gene have been identified [12,18]. The most common are its three polymorphic genotypes *AA*, *AB*, and *BB*, formed by a combination of alleles *A* and *B*, differing in amino acids at positions 136 (Thr → Ile) and 148 (Asp → Ala) [19,20,21,22]. As a result of numerous studies, the tendency to increase protein content in milk from genotype *AA* to *BB* (variant *AB* occupies an intermediate position) has been found [23,24,25]. Moreover, some papers noted the positive effect of the *CSN3* genotype AA on milk heat stability, which is a valuable technological feature in the production and storage of sterilized dairy products, condensed milk (including sweetened), etc. [21,26]. In addition, it has been proved that the *CSN3* gene of *BB* milk, compared to *AA* milk, has better coagulation properties (higher yield of the final product, formation of better gel strength, and shorter duration of protein coagulation) [12,18,26,27]. The *AA* variant of the *CSN3* gene, in turn, is associated with larger micelle sizes and lower gel strength. Most often, these effects are related to the impact of the *CSN3* gene on casein micelle size and the degree of k-casein glycosylation. It is believed that the higher the degree of glycosylation, the more stable the structure of the micelle, while amino acid substitution in position 136 (Thr → Ile) leads to the loss of the glycosylation site for the *BB* variant) [8].

Despite the increased interest in this issue, a scientometric analysis of the studies reveals that most authors focus their attention on the determination of the *CSN3* gene influence during chymosin-induced coagulation of milk proteins as the main method used in the production of 75% of cheeses (of the total production volume) [1]. At the same time, studies evaluating the significance of the *CSN3* genetic marker in the process of acid coagulation of milk proteins are extremely rare in scientific publications [9,28], and there are almost no investigations of the *CSN3* gene influence on the use of mixed variants of milk protein coagulation.

Thus, to fill in the gaps on this topic and to expand the research on the range of cheeses produced, the focus of our work was aimed at establishing the effect of *CSN3* genetic polymorphism on the coagulation ability in acid; rennet; acid–rennet; and heat- and acid-induced as well as heat- and calcium-induced coagulation of milk proteins. To prove the influence of polymorphic variants of *CSN3* on the coagulation ability of milk proteins, we studied skimmed milk, protein-standardized concentrated milk (produced by ultrafiltration (UF), nanofiltration (NF), reverse osmosis (RO)) or by vacuum evaporation (VE) using commercial enzyme preparations containing different amounts of chymosin. Categorization of the obtained research results will make it possible to expand the current amount of molecular genetic studies, formulate further trends in the development of scientific research, and modify the breeding programs to produce high-quality milk suitable to produce a regional range of cheeses in the future.

## 2. Materials and Methods

### 2.1. Design of the Experiment 

**Figure 1:**
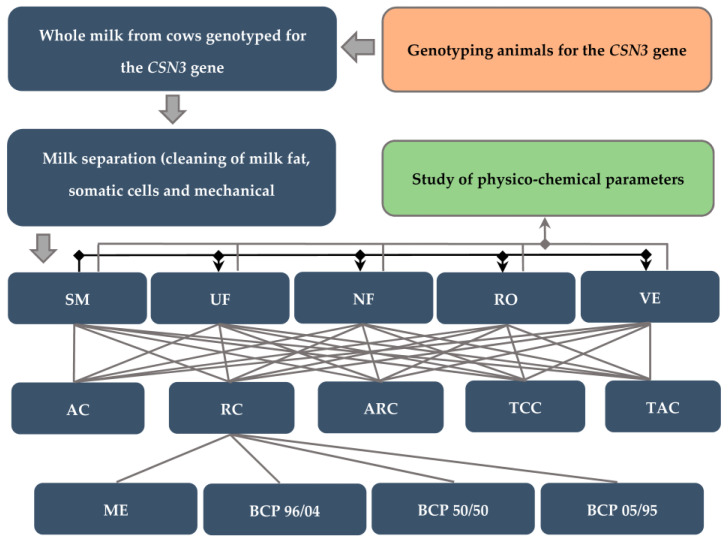
Experiment design. SM—skimmed milk; UF—ultrafiltration retentate; NF—nanofiltration retentate; RO—reverse osmosis retentate; VE—concentrate produced by vacuum evaporation; AC—acid-induced coagulation; RC—rennet-induced coagulation; ARC—acid- and rennet-induced coagulation; TCC—temperature- (heat) and calcium-induced coagulation; TAC—temperature- (heat) and acid-induced coagulation; ME—microbial enzyme (chymosin); BCP 96/04, BCP 50/50, and BCP 05/95—animal enzymes with different ratio of chymosin and pepsin, respectively.

Figure 1 shows the experimental scheme. It includes the following stages of research: a study of the composition of raw milk and its identification by the *CSN3* gene, milk preparation, preparation of retentates and concentrates, analysis of the effects of enzyme preparations, and coagulation methods on the protein cluster.

### 2.2. Milk Samples

Fresh whole cow’s milk was obtained from 353 Black and White breed cows kept on a dairy farm (Lenin State Farm, Russia).

In order to identify raw milk by the *CSN3* gene, preliminary studies were conducted on its allelic ranking by PCR-PDRF analysis. The study of milk and biomaterial (blood) established animals with homozygous (*AA* and *BB*) and heterozygous (*AB*) genotypes by the *CSN3* gene.

### 2.3. Preliminary Milk Preparation

Milk obtained from cows with genotypes *AA*, *AB*, and *BB* according to the *CSN3* gene was delivered to the laboratory within 1 h after milking. Then, it was clarified, heated to 40 ± 5 °C and skimmed by separation of the cream in a laboratory skimmer FJ 90 PP (MilkyDay, Hrdejovice, Czech Republic) to the residual fat content in skimmed milk at 0.05–0.11% (samples CSN3^AA^, CSN3^AB^, CSN3^BB^, respectively). Clarification and skimming of milk are integral and necessary stages of sample preparation to obtain objective data. This is due to the fact that a number of factors (the presence of milk fat, somatic cells, mechanical impurities) are excluded, which can distort the results of studies related to the possibility of using and comparing membrane methods, as well as coagulation methods.

Skimmed milk was pasteurized in the incubator for starter cultures (FlowTech 4/5-10, Them, Denmark) at 72 ± 2 °C with exposure for 20–25 s and cooled to 16 ± 2 °C. Milk was cooled to 4 ± 2 °C and stored until the experiment (no more than 8 h).

### 2.4. Protein Standardization

Skimmed milk was concentrated using the pressure-driven membrane processes (UF, NF, RO) or by VE.

Retentate was obtained at the pilot plant AL 362 (Altair, Vladimir, Russia) with a block of polyethersulfone membrane elements. For UF, membranes with an average pore size of 20 nm and a particle retention threshold of 50 kDa were used (samples of UF-CSN3^AA^, UF-CSN3^AB^, UF-CSN3^BB^ retentates); NF—1.22 nm and 0.6 kDa (samples of NF-CSN3^AA^, NF-CSN3^AB^, NF-CSN3^BB^ retentates); RO—0.1 nm (samples of RO-CSN3^AA^, RO-CSN3^AB^, RO-CSN3^BB^ retentates). Concentration was carried out at 16 ± 2 °C to a mass fraction of the total protein of 6.6% (on average).

The vacuum evaporation of skimmed milk was carried out on a laboratory single-effect evaporator (VNIMI, Russia) at 70 ± 2 °C, a vacuum of 0.09 MPa, and 2.1-fold concentration in evaporated moisture (samples of VE-CSN3^AA^, VE-CSN3^AB^, VE-CSN3^BB^ concentrates) to the total protein of 6.6% (on average).

For comparability of the evaluation criteria, retentates and concentrates were adjusted (if necessary) with permeates and distilled water (respectively) to the protein content of 6.6%. This operation was included because the protein profile plays an essential role in the gel strength under the action of milk-converting enzymes [29,30,31].

### 2.5. Samples of Coagulating Enzymes

To study the coagulation properties of dairy systems, enzyme preparations of various compositions and specificities were used:

- Milk-clotting enzyme of microbial origin (chymosin) Microclerici 2400 IMCU g^−1^ (Sacco Sistem, Cadorago, Italy), obtained in the process of controlled cultivation of the strains *Rhizomucor miehei*—ME;
- A mixture of beef chymosin with pepsin Clerici 96/04 (Sacco Sistem, Cadorago, Italy) with an activity of 2400 IMCU g^−1^—BCP 96/04;
- A mixture of beef chymosin with pepsin Clerici 50/50 (Sacco Sistem, Cadorago, Italy) with an activity of 800 IMCU g^−1^—BCP 50/50;
- A mixture of beef chymosin with pepsin 05/95 (LLC “Modern Technologies”, Moscow, Russia) activity 800 IMCU g^−1^—BCP 05/95.

For comparability of the results, the activity of all enzymes was recalculated to 800 IMCU g^−1^.

### 2.6. Chemical and Physical Analyzes

Standardized and generally accepted methods of chemical and physical control of dairy products were used for research. The total solids content was determined by the control thermogravimetric method (Method 6731; ISO, 2010). The fat content was conducted using the Gerber method (Method 19662; ISO, 2018; Method 11870; ISO, 2009). In order to determine the protein content measuring of the total nitrogen by the Kjeldahl method on the protein analyzer, Kjeltec-2400 Auto Analyzer (Foss Electric, Hillerød, Denmark) with its subsequent conversion to protein using a conversion factor of 6.38 (Method 1871; ISO, 2009; Method 8968-1; ISO, 2014) was used. Non–protein nitrogen was conducted using precipitation of protein components of samples with trichloroacetic acid, followed by measurement of total nitrogen in the filtrate, as described above. Casein and whey proteins were analyzed by the reference method (Method 17997-1; ISO, 2004) using acid precipitation of casein and measurement of total nitrogen in the filtrate. Then the ratio of whey proteins and casein fractions was calculated using the obtained data on the content of total and non–protein nitrogen. Milk protein composition was conducted using reversed-phase HPLC according to the method [6]. Lactose was determined using the enzymatic method based on pH difference (Method 26462; ISO, 2010). Titratable acidity was analyzed by titration 0.1 N NaOH in the presence of 1% alcohol solution of the phenolphthalein indicator and expressed in Turner degrees (°T); pH was analyzed by the potentiometric method using a laboratory pH meter inoLab pH Level 1 (WTW, Weilheim, Germany); and ash content was analyzed by burning dried samples at 550 °C in a muffle electric furnace (MP–2UM, Utena, Lithuania) [32]. The concentration of calcium, magnesium, sodium, and potassium was determined using inductively coupled plasma optical emission spectroscopy (ICP-OES) with Agilent 5110 ICP-OES instrument (Agilent Technologies Bayan Lepas Free, Penang, Malaysia). The content of phosphates and citrates was measured by capillary electrophoresis on a Kapel-205 device (Lumex-Marketing, Saint-Petersburg, Russia).

Evaluation of the polymorphism of milk proteins by the *CSN3* gene was performed by PCR-PDRF analysis of raw milk according to the method [33]. A total of 1.5 mL of the analyzed samples in Eppendorf-type tubes was centrifuged at 9800× *g* for 15 min using a CM-50 mini-centrifuge (Elmi, Riga, Latvia). The supernatant was removed with separate 200 µL tips without a filter using an aspirator with a flask trap FTA-1 (Biosan, Riga, Latvia). DNA was isolated using a set of “DNA-sorb-S-M” (Central Research Institute of Epidemiology, Russia), according to the instructions for the set. PCR was performed in 20 µL of a reaction mixture, including 13 µL of dH_2_O, 2 µL of dNTP, 2 µL of SE buffer, and 0.2 µL of 1 unit. Taq DNA polymerase, 2 µL DNA samples, and 0.4 µL 0.5 µm primers JK5 and JK3 provided amplification of the PCR product with a length of 350 bp, under thermal cycling mode: ×1: 94 °C–4 min; ×35: 94 °C–10 s, 63 °C–10 s, 72 °C–10 s; 72 °C–7 min. The PCR samples obtained were treated with 5 units of Hinf I restriction endonuclease at 37 °C for 12 ± 2 h. The following genotype-specific PCR-PDRF fragments were formed: *AA* = 134/131/85 bp, *BB* = 265/85 bp, and *AB* = 265/134/131/85. Their detection was carried out using electrophoresis in a 2% agarose gel using ethidium bromide to visualize DNA in a UV transilluminator.

### 2.7. Determination of the Size of Casein Micelles

The size of casein micelles was determined using the LS 13 320 XR laser diffraction particle size analyzer (Beckman Coulter, Indianapolis, IN, USA) with a measurement range from 10 nm to 3500 µm. The analysis was based on the principle of light scattering supplemented by PIDS (polarization intensity differential scattering) technology. The samples were preliminarily defatted with hexane. For this purpose, the sample and hexane in a 1:1 ratio were added to centrifugation tubes. The contents were stirred for 1 min and centrifuged at 2400× *g* for 5 min. The surface layer of hexane with fat was removed using automatic pipettes with disposable tips. The defatted samples were washed twice with deionized water at 60 °C under the same conditions to remove hexane residues.

### 2.8. Determination of Coagulation Ability

The coagulation properties of milk, retentates, and concentrates were analyzed by changing the dynamic viscosity (η, cPs) on a DV-II+Pro rotary viscometer (Brookfield, Middleboro, MA, USA) with a fixed external cylinder. SC4-13R(P) sample chambers made of a material with high thermal conductivity were used to ensure accelerated heating and a rotating measuring rod with a spindle SC4-3. The choice of a spindle with a conical shape of the lower end is due to the need to level the difference in shear rates depending on the distance to the axis of rotation. Before the experiment, each sample in a volume of 10 mL was thermostated in a viscometer chamber on a water bath at a temperature of 40 ± 1 °C for at least 10 min. Then 0.1 mL of a 10% solution of one of the enzyme preparations (activity was recalculated by 800 IMCU g^−1^) was added to the chamber and quickly mixed. Within 15 ± 2 s after the introduction of the enzyme preparation, the sample was placed in a thermostat fixed on the device with a temperature of 40 ± 1 °C. The rotation was started with a minimum constant spindle rotation speed of 10 min^−1^. The dynamic viscosity indicators were recorded for 7 ± 2 min. For each of the samples, dependencies were plotted in the range of dynamic viscosity from 0 to 2000 cPs.

In parallel with the study of dynamic viscosity, 500 mL samples were prepared using 16.7 mL of a 1% solution of one of the enzyme preparations and thermostated in a dry-air cabinet (TSO-1/80 SPU, Russia) at a temperature of 40 ± 1 °C for 180–200 min until a visually needed gel strength was reached. The clot was cut into cubes of 10 × 10 mm and left intact. The whey was separated by filtration through dacron (PET) material.

The protein conservation coefficient (K) in the clot was calculated as follows:

K = (A × B)/(C × 100)(1)

where A is the clot yield, %; B is the protein in the clot, %; and C is the protein in milk before coagulation, %.

### 2.9. Methods of Milk Protein Coagulation

The following types of biochemical methods of protein destabilization (coagulation) were selected for the study:

- Acid coagulation (AC)—a decrease in the pH value below the isoelectric point of the casein complex (4.6–4.7) due to lactic acid formed as a result of fermentation of lactose during fermentation of skimmed milk or retentates or concentrates by mesophilic and thermophilic lactic acid microorganisms (*Lactococcus lactis* strains 79_5_, 79_10_, 79_13_ and *Streptococcus thermophilus* strain 6 kb) in an amount of 5% of the mass of fermented milk;
- Rennet coagulation (RC)—protein coagulation exposed to 16.7 mL of 1% solution of one of the enzyme preparations (ME, BCP 96/04, BCP 50/50, BCP 05/95), the activity of which was recalculated to 800 IMCU g^−1^;
- Acid–rennet coagulation (ARC)—combined protein coagulation exposed to 16.7 mL of a 1% solution of an enzyme preparation (BCP 96/04) and lactic acid formed as a result of fermentation of dairy systems by mesophilic and thermophilic lactic acid microorganisms (*Lactococcus lactis* strains 79_5_, 79_10_, 79_13_ and *Streptococcus thermophilus* strain 6 kb) in an amount of 5% of the mass of fermented milk;
- Heat- and calcium-induced coagulation (TCC)—protein coagulation under the action of a 15% solution of strong electrolyte CaCl_2_ in combination with high-temperature treatment 92 ± 2 °C for 5.0 ± 0.1 min;
- Heat- and acid-induced coagulation (TAC)—protein coagulation under the action of 80% lactic acid (up to pH 5.4) in combination with high-temperature treatment 92 ± 2 °C.

### 2.10. Statistical Analysis

All experiments were performed independently in triplicate. The results were processed by analysis of variance (ANOVA) with subsequent comparison of average values according to Student’s *t*-test using Statistica 12.0 software with a significance level of 95%. All the results were presented as the mean (±) standard deviation (SD).

## 3. Results and Discussion

### 3.1. Alleles and the Genotype Frequency for the CSN3 Locus

**Figure 2:**
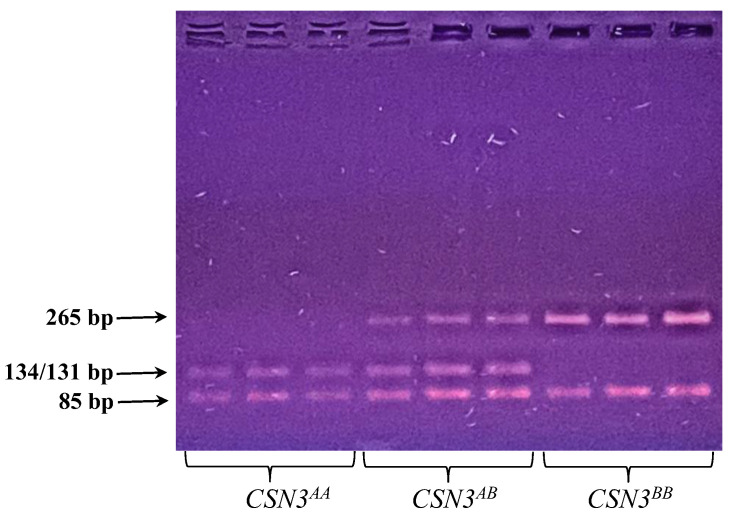
PCR-PDF analysis of raw milk samples.

In order to identify raw milk from the *CSN3* gene obtained from black-motley breed cows, preliminary studies were performed on its allelic ranking by PCR-PDRF analysis (Figure 2). The polymorphic regions of the *CSN3* gene corresponded to genotypes AA (131(134)/85 bp), AB (265/131(134)/85 bp), and BB (265/85 bp).

Three hundred and fifty-three cows of the *CSN3* gene were genotyped by DNA analysis of the biomaterial; 225 of them were identified with homozygous genotype *AA*, 31 with genotype *BB* and 97 cows with heterozygous genotype *AB*. Thus, the frequency of genotypes in the black-motley cows was 63.7% for *AA*, 27.5% for *AB*, and 8.8% for *BB*. The frequency of the *A* allele of the *CSN3* gene was 77.5%, and that of the *B* allele was 22.5% (Table 1). The obtained distribution, taking into account insignificant deviations typical for individual farms, in general, agrees with the earlier studies [34].

In order to evaluate the effect of *CSN3* gene polymorphism on milk productivity and the physicochemical composition of milk, a block of studies was conducted. The average results of the studies are presented in Table 2.

Analysis of the productivity of polymorphic animals for the *CSN3* gene showed that allele A influenced higher average milk yield during 305-day lactation compared to individuals with allele *B*. The difference in cow productivity between homozygous genotypes *AA* and *BB* was 376 kg of milk per lactation period. However, no significant differences were found between genotypes *AA* and *AB* for the *CSN3* gene (the difference in milk yield was 62 kg). Milk obtained from cows with the *BB* genotype was characterized by higher fat content (3.88%) and total fat yield (387.3 kg). Moreover, for genotype *BB*, a higher protein (3.30%) was observed. At the same time, the total protein yield was lower than that of cows with the *AA* genotype. These results confirm previous studies conducted [18,24].

### 3.2. Quantitative and Qualitative Composition of Skim Milk, Retentates and Concentrates Depending on the Polymorphism of the CSN3 Gene

Table 3 presents the physico-chemical composition of skimmed milk, retentates, and concentrates. There is a trend in the difference in the physico-chemical parameters of skimmed milk, retentates, and concentrates depending on the polymorphism of the *CSN3* gene. As expected, the concentration of the protein content and casein fractions in CSN3^BB^ ((3.39 ± 0.10)% and (2.71 ± 0.14)%) was higher than in CSN3^AA^ ((3.30 ± 0.12)% and (2.58 ± 0.09)%) and CSN3^AB^ (3.24 ± 0.09% and 2.53 ± 0.14%). Consequently, the content of whey proteins is lower (0.68 ± 0.05%, 0.72 ± 0.07%, 0.71 ± 0.05%, respectively), which correlates with the results of Djedović, R. et al., Akkerman, M. et al. and Uniacke-Lowe, T. et al. [18,35,36]. The use of various methods of concentration of skimmed milk until the value of total protein of 6.6% did not distort the detected trend in relation to casein and whey fractions. However, a decrease in the efficiency of the process was observed in the case of using UF, which occurred because of the membrane element’s selective permeability to micellar casein proteins. The transition of casein micelles through a semipermeable membrane is most likely related to their smaller average size, characteristic of milk obtained from cows with the *CSN3* gene allele *B* compared to the A allele [20,26]. Some damage to casein micelles caused by transmembrane pressure and shear force on the membrane surface could also be the reason for this [37,38,39,40]. Additionally, selective membrane permeability is responsible for the pattern of increased lactose and salt content in retentates, and hence total solids compared to the initial skimmed milk (RO ˃ NF ˃ UF) [41,42]. Moreover, milk with the *CSN BB* genotype was characterized by higher calcium content and lower phosphate and citrate content compared to the *CSN3 AA* and *CSN3 AB* genotypes. This may positively affect the suitability of milk for cheese production and reduce the heat stability of milk in the production of sterilized and canned dairy products [27,43].

Similar values of the total solids, lactose, and salt content were observed in RO-retentates and VE-concentrates. This could be due to the removal of only water molecules and some mineral ions from the milk system for RO and only free moisture in the form of water vapor with a small fraction of native aromatics and low-molecular-weight volatile fatty acids for VE [44,45].

A regular increase in the titratable acidity values in all the concentrated samples was observed in relation to the milk. Titratable acidity values for RO and VE were higher than for UF and NF, which was caused in the first case by removing only water from the milk system and in the second case by the transition of a part of the organic acids into the permeate. For the same reasons, the pH values for RO and VE, as well as for UF and NF, were close to each other (respectively).

**Figure 3:**
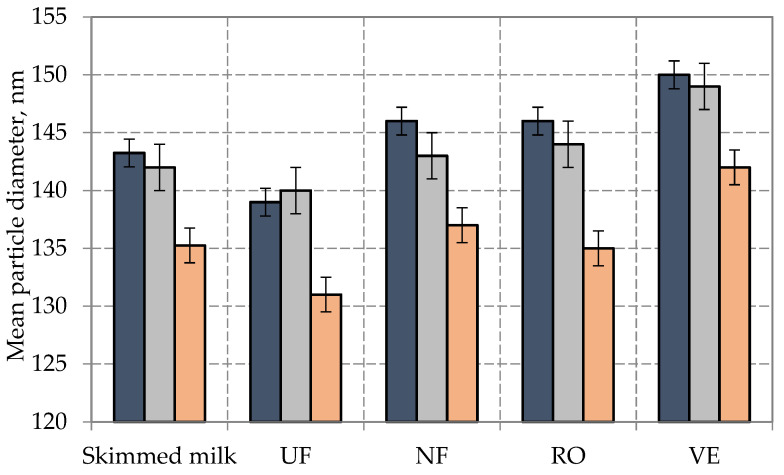
Average sizes of casein micelles in milk obtained from cows with genotypes *AA*, *AB*, and *BB* ac-cording to the *CSN3* gene and after its concentration (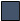-CSN3^AA^; 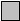-CSN3^AB^; 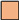-CSN3^BB^). Error bars represent standard errors.

To confirm assumptions related to the dependence of milk genotype and average casein micelle size [20,26,35], as well as aggregation of protein particles and damage of casein micelles during the pressure-driven membrane processes [37,38,39,40], analyses of average particle size were carried out. Due to the preliminary preparation of samples by their additional defatting, representative data were obtained, enabling comparison of the average sizes of protein particles of the samples under study with the use of PIDS technology (polarization intensity differential scattering technology) and laser diffraction (Figure 3).

The average size of casein micelles in CSN3^AA^ was 143 nm; in CSN3^AB^, it was 42 nm; and in CSN3^BB^, it was 135 nm, which confirmed the results of Bijl E. et al., Frederiksen P.D. et al., and Akkerman M. et al. [20,26,35] on the correlation of genetic variants *A* and *B* of k-casein with an average size of casein micelles in milk. The pressure-driven membrane processes had a slight effect on the average size (there was a decrease of 2–4 nm for UF and an increase of only 1–2 nm for NF and RO), which may be due to possible partial destruction or conformational change in protein clusters [37,38,39,40]. An increase in the average diameter (by 6–7 nm) was identified in all the concentrated samples (VE-CSN3^AA^, VE-CSN3^AB^, VE-CSN3^BB^) due to some aggregation of protein particles during vacuum evaporation [46,47].

Since the values of the quantitative and qualitative composition of all samples obtained from milk from cows with *AB* genotypes according to the *CSN3* gene were closest to *AA*, further studies were carried out only for *AA* and *BB*.

### 3.3. The Effect of Enzyme Preparations on the Milk Protein Cluster

The coagulation of casein, being the main technological stage in the production of cheese, cottage cheese, casein, and some other dairy products, is mainly caused by rennet or its substitutes. At present, microbial chymosin, beef chymosin, and various mixtures of chymosin with pepsin in different ratios, as well as beef pepsin, are used [10,29,30,48,49,50]. Given the current knowledge of biotechnology, enzymes are fairly well understood and predictable, especially in comparison to milk, the substrate of the rennet clotting process. Practice shows that it is possible to obtain a standard enzyme, but obtaining standard milk is still a problematic and challenging process. A potential solution to standardize the behavior of enzymes of all types during milk clotting could be the study of the raw milk protein with the determination of the genotype of the lactating animal by k-casein.

**Figure 4:**
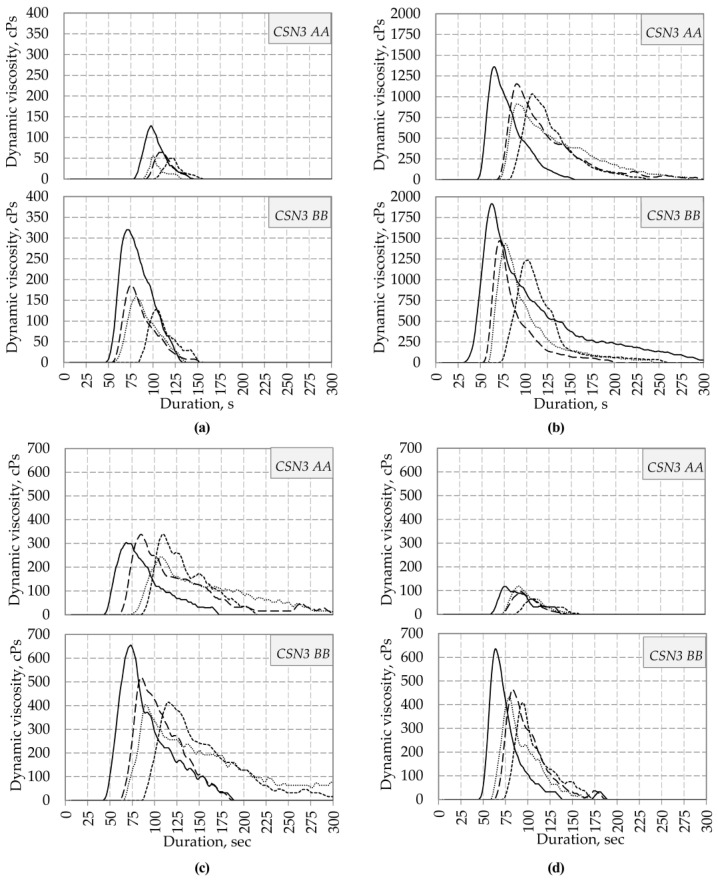

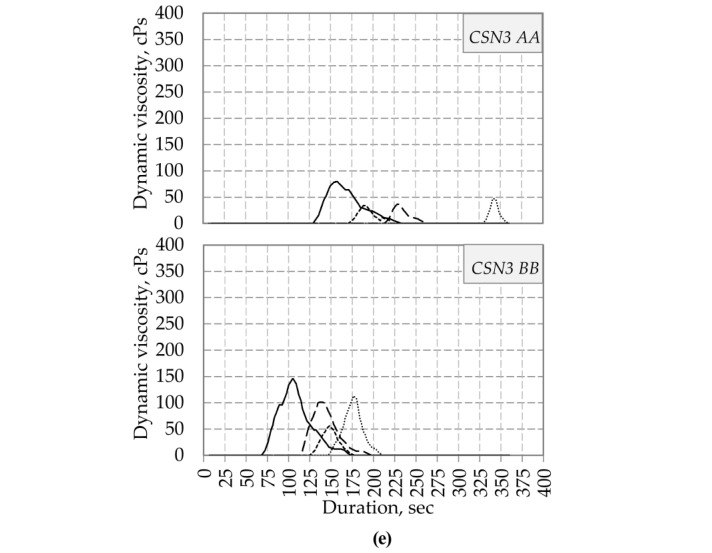
Rheograms of coagulation properties of samples depending on the type of enzyme preparations and *CSN3* polymorphism ((**a**) skimmed milk; (**b**) UF; (**c**) NF; (**d**) RO; (**e**) VE; 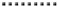
—ME; 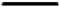
—BCP 96/04; 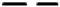
—BCP 50/50; 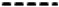
—BCP 05/95).

Figure 4 presents the results of the analysis of coagulation properties of skimmed milk, retentates, and concentrates depending on the type of enzyme preparations and the *CSN3* gene polymorphism. This analysis was performed to identify reference points characterizing the milk-clotting activity of enzyme preparations. The resulting curve configurations reflected changes in dynamic viscosity parameters and corresponded to typical rennet coagulation rheograms. The rheograms clearly show all the stages characterizing the gelation process: zero dynamic viscosity during the induction period (lag phase); an increase in dynamic viscosity to the peak of gelation (mass coagulation); maximum dynamic viscosity (gel point); and a decrease in dynamic viscosity during the destruction of the clot to the initial zero values (syneresis) [51,52,53,54]. The rheograms presented in Figure 4a clearly show a trend of the predominance of coagulation ability in CSN3^BB^ compared to CSN3^AA^, both in coagulation rate (by 20–25%) and increase in gel strength (by 2.5–3 times). This correlates with the data [21]. BCP 96/04 has the best milk-clotting activity, regardless of the genotypic affiliation of milk [55]. At the same time, in all the studies, there was a slowdown in the rate of clot and gel strength formation with a decrease in chymosin in the enzyme preparation. A comparative evaluation of rheograms of the samples using microbial and animal chymosin revealed the best milk-clotting activity of BCP 96/04 in relation to k-casein and, consequently, casein micelles in general. The rheograms shown in Figure 4b indicate the better coagulation ability of UF-CSN3^BB^ compared to UF-CSN3^AA^ when using all types of the studied enzymes. The duration of the lag phase decreased by 1.2–1.6 times, and the maximum dynamic viscosity increased by 20–35%. A comparative analysis of substrate–enzyme interactions showed that BCP 96/04, compared with other preparations, has an increased rate of a k-casein hydrolysis reaction. This is characterized by a minimum duration of induction with the formation of a better gel strength (for UF-CSN3^AA^ by 15–50%, for UF-CSN3^BB^ by 31–58%). Distinct dependence of the gel strength of NF-retentates treated by various enzyme preparations on the genetic markers of milk for the *CSN3* gene was revealed (Figure 4c). The clot samples obtained from the milk of cows with the *CSN3 BB* genotype differed from the *CSN3 AA* samples by an increased dynamic viscosity at the peak of gelation by 1.6–1.9 times. At the same time, no significant differences were observed in the duration of the induction period between NF-CSN3^AA^ and NF-CSN3^BB^ for the entire range of enzymes studied.

The kinetic pattern of gelation of the NF-CSN3^BB^ sample showed that the best structural and mechanical properties were ascertained in a clot obtained using BCP 96/04. The values of its dynamic viscosity at the gel point exceeded the values of BCP 50/50 by 27%, BCP 05/95 by 59%, and ME by 62%. Figure 4d shows that the intensity of the first stage of coagulation in RO-CSN3^BB^ using all the studied enzymes is higher than in RO-CSN3^AA^, as well as the coagulation rate and the values of rheological parameters at the stage of maximum clot structure formation are significantly higher. The maximum coagulation effect was achieved in the RO-CSN3^BB^ sample using BCP 96/04, the values of the dynamic viscosity of which amounted to 640 cPs. A comparative evaluation of rennet coagulation rheograms (Figure 4e) indicates an average two-fold increase in the duration of the onset of the mass coagulation stage in VE-CSN3^AA^ for all types of enzyme preparations used. At the same time, there is a tendency for peak values to prevail at the points of gelation for VE-CSN3^BB^ compared to VE-CSN3^AA^ (by two times on average). The best coagulation ability in the samples obtained using vacuum evaporation of milk of both VE-CSN3^AA^ and VE-CSN3^BB^ was ascertained by the enzyme preparation BCP 96/04 (75 and 149 cPs, respectively).

By comparing the parameters of rheograms of unconcentrated and concentrated samples, it was found that in the optimal mode, the gelation process took place only in UF-CSN3^BB^. This is accounted for by the largest amount of casein due to the degree of concentration while reducing the average distance between casein micelles [54,56,57]. Thus harder gels are obtained due to higher pH values and the content of soluble calcium phosphate due to solubilization and maintaining close values of lactose content, as in the original milk [45,57]. This shows a positive effect on the coagulation of milk allele B in the k-casein [26,58].

Table 4 presents data from analysis of the physico-chemical parameters of clots of skimmed milk samples and UF, NF, and RO retentates depending on *CSN3* polymorphism. The samples obtained from skimmed milk CSN3^BB^ had a higher clot yield relative to CSN3^AA^. The clots of these samples were characterized by a high content of solids and protein. Comparison of the milk-clotting activity of the enzyme preparations under study revealed a tendency for maximum complete protein extraction when using BCP 96/04 regardless of substrate genotype (CSN3^AA^—0.784; CSN3^BB^—0.821). Evaluation of the quantitative indicators of clots from UF-CSN3^AA^ and UF-CSN3^BB^ showed that the highest clot mass was obtained using UF-CSN3^BB^, regardless of the type of milk clotting enzymes. The lowest clot yield with the highest total solids and protein content was observed in UF-CSN3^AA^ and UF-CSN3^BB^ coagulated by BCP 96/04. The highest value of the protein retention factor was observed (0.914) with rennet coagulation of UF-CSN3^BB^ with the enzyme preparation BCP 96/04. Gravimetric analysis of the mass of NF-CSN3^AA^ and NF-CSN3^BB^ clots demonstrated a similar pattern of the predominance of yields in NF-CSN3^BB^ over NF-CSN3^AA^ when comparing the types of enzyme preparations. Considering the effect of enzyme preparations on concentrated dairy systems within genetic groups, the maximum values for the total solids content and protein were reached in the clots using BCP 96/04. NF-CSN3^BB^, coagulated by BCP 96/04, had the highest value of the protein retention factor in the clot (0.921). The predominance of the yield of clots from RO-CSN3^BB^ over RO-CSN3^AA^ was also established in the RO-retentates analysis. The maximum values of the total solids content were determined in RO-CSN3^AA^ and RO-CSN3^BB^ (28.66% and 28.73%, respectively). The maximum protein content (21.11% and 21.07%, respectively) was reached using BCP 96/04. Furthermore, these samples had the highest values of protein retention factor in the clot: 0.918 (RO-CSN3^AA^) and 0.932 (RO-CSN3^BB^).

Analysis of the coagulation ability of VE-CSN3^AA^ and VE-CSN3^BB^ under the influence of all the enzyme preparations used in this experiment showed their extremely low efficiency in destabilizing casein micelles (formation of a gel strength with at least a small degree of syneresis was not observed). This is accounted for by the partial integration of k-casein with β-lactoglobulin, accompanied by an increase in the size and hydrophilicity of casein micelles during milk concentration by vacuum evaporation [47,59]. As a result, it was not possible to conduct a physico-chemical analysis of these clots.

**Figure 5:**
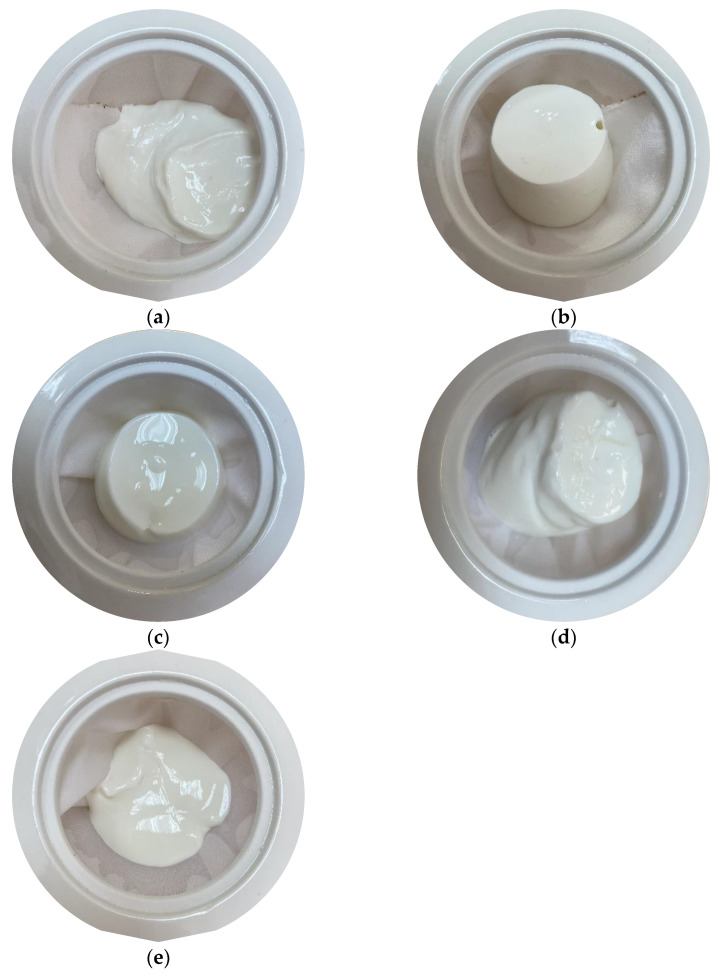
Examples of the appearance of clots obtained by coagulation of samples of skimmed milk, retentates, and concentrate with the enzyme preparation BCP 96/04 ((**a**) CSN3^BB^; (**b**) UF-CSN3^BB^; (**c**) NF-CSN3^BB^; (**d**) RO-CSN3^BB^; (**e**) VE-CSN3^BB^).

Since a comprehensive assessment of the coagulation properties of all samples revealed the best milk-clotting ability of the enzyme preparation BCP 96/04, Figure 5 shows a visualization of the appearance of clots obtained by coagulation of skimmed milk, retentates, and concentrate samples from milk from cows with the *BB* genotype by the *CSN3* gene.

The data presented in Figure 4 and Table 4 confirmed the previously obtained patterns of the positive effect of the *BB* genotype on the *CSN3* gene on the coagulation properties of not only milk but also the retentates obtained from it [23,26]. In all samples produced from milk obtained from cows with the *CSN3 BB* genotype, the duration of coagulation had the lowest values, while the viscosity during the formation of clots was the highest.

The rheological studies of the gelation kinetics revealed the greatest effectiveness of the use of BCP 96/04 in relation to other enzyme preparations used. This fact is associated with the formation of a denser clot in the case of enzyme preparations with a predominance of beef chymosin and a looser clot, accompanied by a higher degree of protein transition to whey when using pepsin. This is due to the specific proteolytic (milk-clotting) activity of chymosin and pepsin in relation to the protein fraction of k-casein, consisting of their ability to hydrolyze the peptide bond at the amino acid site between phenylalanine (Phe) and methionine (Met) at position 105-106. The main chymosin has only specific proteolytic activity, while pepsin has ambivalent activity (both specific and non-specific). This can lead to the rupture of not only the peptide bond in the k-casein between Phe105 and Met106 but also other peptide bonds of all casein fractions. It can thus lead to the formation of low molecular weight peptides that impart products (cottage cheese, cheese etc.) a bitter taste [36,60,61,62].

Thus, the impact of enzyme preparations different in composition and specificity on the coagulation properties of milk produced from cows genotyped for the *CSN3* gene has been revealed. The best coagulation properties (the duration of the induction period, the period of mass coagulation, and the dynamic viscosity at the gel point) with optimal values of clot yield, protein content, and protein retention factor in the clot were identified in the samples of UF-retentate produced from milk obtained from cows with the *CSN3 BB* genotype, using an enzyme preparation consisting of beef chymosin (96%) and pepsin (4%).

### 3.4. The Effect of CSN3 Polymorphisms on the Technological Properties of Biotechnologically and Chemically Induced Milk Gels

The production of cottage cheese products and cheeses is based on the deliberate break-down of colloidal dairy systems by destabilizing casein micelles under the influence of various factors, such as organic and inorganic acids, milk-converting enzymes, high temperatures, etc. Classical and innovative production technologies for this group of products imply the use of not only one of the types of destabilizing factors but also their combination [7].

Table 5 presents data on the physico-chemical parameters of the clots of skimmed milk samples, retentates, and concentrates obtained under the influence of various biochemical and chemical methods of protein destabilization (coagulation), depending on the polymorphism of *CSN3*. Clots from CSN3^AA^ during AC had a 2.3% lower yield of the product than from CSN3^BB^, although the protein mass in the clot of the samples was practically equal within the measurement accuracy of the method. With ARC, this trend continued (the clot yield is 1% lower). However, the protein mass in the CSN3^BB^ clot increased by 8.9%. TCC analysis identified a slight increase in the clot yield in CSN3^BB^ by 0.4%, while there were no significant deviations in the absolute masses of protein in the clots. The opposite pattern was observed in the analysis of clots’ yield obtained by TAC. The yield of clots from CSN3^BB^ was 4.1% lower than from CSN3^AA^, although the protein mass in the CSN3^BB^ clot was 3.7% higher. Analysis of physicochemical parameters and mass transitions of UF-CSN3^AA^ clots in relation to UF-CSN3^BB^ obtained during AC and TCC demonstrated a similar dependence (comparing with the data of CSN3^AA^ and CSN3^BB^ clots) of a higher clot yield in UF-CSN3^BB^. The maximum yields were determined using TCC (UF-CSN3^AA^—37.3%, UF-CSN3^BB^—41.5%). There were no correlating differences in clot yields in UF-CSN3^AA^ and UF-CSN3^BB^ induced by ARC and TAC. However, the protein mass in the UF-CSN3^BB^ clot was 2.1% higher for ARC and 1.8% lower for TAC than in UF-CSN3^AA^. This is due to various factors, such as partial decalcification of retentate in the UF process, the smaller average size of casein micelles in UF-CSN3^BB^, and change in the ratio of protein fractions, which together leads to varying degrees of denaturation of whey proteins on the surface of casein micelles with their subsequent coagulation [20,63,64,65]. The dependences for UF-CSN3^AA^ and UF-CSN3^BB^ were kept to a different extent for clots of NF-retentate samples. In the case of TCC, the maximum values of the clot yield established (for NF-CSN3^AA^—38.3%, NF-CSN3^BB^—41.3%) were comparable with similar samples obtained from UF-retentates, while in the case of TAC, the largest protein masses in the clot were obtained (for NF-CSN3^AA^—6.45 g, NF-CSN3^BB^—6.34 g). It was also found that the maximum yields of clots of RO-retentate samples were observed using TCC (RO-CSN3^AA^—38.0%, RO-CSN3^BB^—40.9%). These were comparable with samples of UF-retentates and NF-retentates. The maximum yield was observed at AC and ARC of VE-CSN3^AA^ and VE-CSN3^BB^ clots. However, with these methods of coagulation, a low level of syneresis was noted in the VE concentrate clots, ultimately affecting their consistency (smearing) and a decrease in protein content. This fact confirms the low reactivity of the enzyme preparation with respect to the k-casein of VE-concentrated samples (Figure 4e). This evened out the difference between AC and ARC. The samples obtained with TCC (for VE-CSN3^AA^—6.01 g, VE-CSN3^BB^—5.92 g) and TAC (for VE-CSN3^AA^—6.18 g, VE-CSN3^BB^—6.05 g) had the highest values of the protein masses in the clots. A comparison of RC clot yields using BCP 96/04 (Table 4) obtained from skimmed milk and concentrated samples with other methods of coagulation (Table 5) revealed their similarity to ARC.

**Figure 6:**
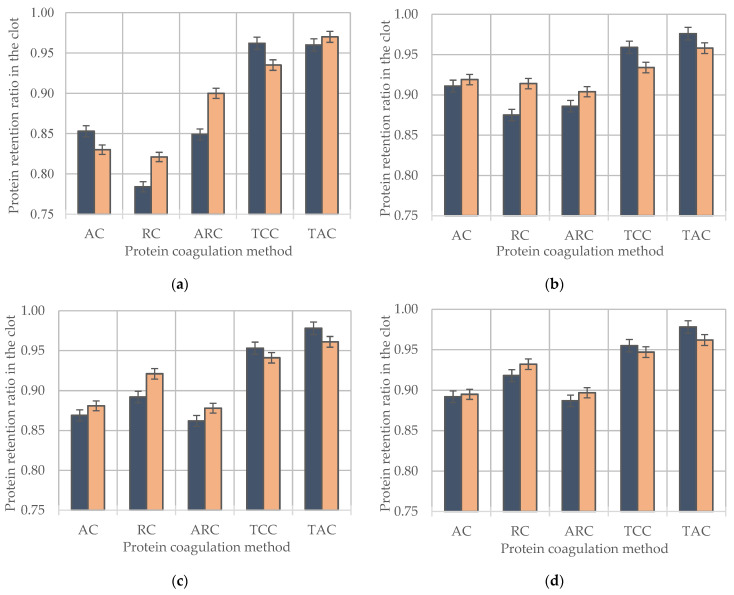

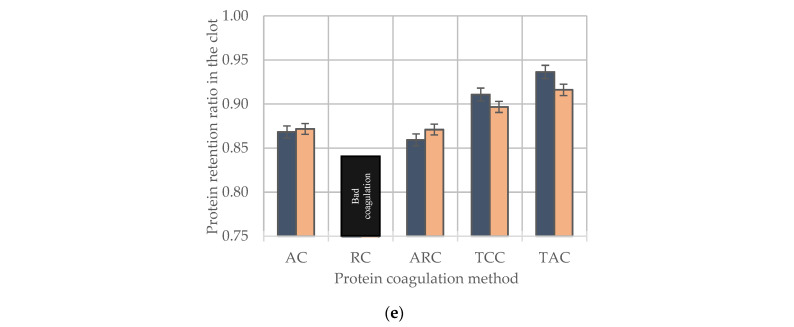
Comparative evaluation of protein preservation coefficients in clots depending on *CSN3* polymorphism, milk system concentration methods, and coagulation methods ((**a**) Skimmed milk; (**b**) UF; (**c**) NF; (**d**) RO; (**e**) VE). 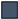-*CSN3 AA*; 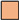-*CSN3 BB*. Error bars represent standard errors.

The technological potential of milk is related to the coagulation ability of the protein cluster when affected by a combination of acid, rennet, and heat treatments. Due to this fact, an assessment of the transition of protein into a clot, namely the protein retention factor (Figure 6), was carried out to fully monitor the cumulative effect of *CSN3* genetic polymorphism and pressure-driven membrane processes on the formation of the technological potential of milk. The positive effect of the *CSN3 BB* genotype on the protein retention factor in AC and ARC clots of retentate samples (UF, NF, RO) and also in skimmed milk clots in the RC was observed. At the same time, UF-retentate subjected to RC compared to non-concentrated milk forms better gel strength, which corresponds with the study [66]. In the samples induced by AC, the maximum values of the protein retention factor were observed in UF retentates for both CSN3^AA^ and CSN3^BB^ (0.911 and 0.919, respectively), and for ARC, it was 0.886 (CSN3^AA^) and 0.904 (CSN3^BB^). The higher value of the protein retention factor in clots CSN3^BB^ of retentates of all types of pressure-driven membrane processes is associated with the predominance of casein micelles with a smaller average diameter. This increases the reactivity of the hydrolysis of k-casein exposed to milk-clotting enzyme preparations (with rennet and acid–rennet coagulation) [20,26]. A decrease in the average diameter of casein micelles is also accompanied by a decrease in their total negative charge. Under the action of lactic acid (at AC and ARC), this leads to accelerated destabilization of micelles (due to a decrease in steric and electrostatic stabilization caused by the destruction of the outer layer of the surface of casein micelles consisting of k-casein) and the formation of a better gel strength due to increasing the number of connections between micelles [14,15,16]. Analysis of the data obtained with TCC and TAC revealed opposite patterns. They consisted of the more efficient protein retention in the clots of UF-CSN3^AA^, NF-CSN3^AA^, and RO-CSN3^AA^. The values of the protein retention factor in the clots of retentates subjected to TCC were characterized for samples of UF-CSN3^AA^, NF-CSN3^AA^, and RO-CSN3^AA^ by the highest values (0.959, 0.953, and 0.955, respectively) in relation to UF-CSN3^BB^, NF-CSN3^BB,^ and RO-CSN3^BB^ (0.934, 0.941, and 0.947, respectively). Evaluation of the data related to TAC shows an equivalent highest efficiency of the protein extraction process regardless of the method of concentration (0.976, 0.978, and 0.978 for UF-CSN3^AA^, NF-CSN3^AA^, and RO-CSN3^AA^, respectively).

The results obtained (in the case of using TCC) can be explained by a decrease in the negative charge of the surface of casein micelles and the associated repulsion between protein particles under the action of positively charged ions of divalent calcium. As a result, the protein hydration layer becomes thinner, its heat stability decreases, and complex coagulation occurs. It is not only casein that coagulates but also denatured whey proteins associated with it, causing the highest clot yield compared to other coagulation methods [67,68]. This process is more strongly manifested during the coagulation of CSN3^AA^ samples. This is due to the presence of protein particles of a larger average diameter in them with respect to CSN3^BB^ samples. TAC is also characterized by a high degree of protein fraction extraction due to the precipitation of whey proteins together with casein. A decrease in the pH below the normal value of milk under heat exposure leads to the adsorption of whey proteins on the surface of casein micelles, thereby causing an increase in their diameter. In the case of CSN3^AA^ samples, this resulted in an even greater increase in micelles [69,70,71].

## 4. Conclusions

The study confirmed a pattern, according to which milk from animals with the *AA* and *AB* genotype according to the *CSN3* gene had a larger average size of casein micelles compared to the *BB* genotype. At the same time, UF was found to have a more significant effect on reducing the size of protein particles than NF and RO. VE, on the contrary, led to the insignificant aggregation of protein particles. The influence of enzyme preparations of different compositions and specificity on the coagulation properties of skimmed milk, retentates, and concentrates obtained from milk from cows genotyped by the *CSN3* gene was determined. UF retentates produced from milk obtained from cows with *CSN3 BB* genotype using an enzyme preparation consisting of 96% beef chymosin with 4% pepsin added had the best coagulation properties (duration of induction period, mass coagulation period, dynamic viscosity in gel point) with optimal values of clot yield, protein content and protein retention factor in the clot. The correlation between the *CSN3 BB* genotype and destabilization (coagulation) of proteins of skimmed milk, retentates, and concentrates obtained from milk from cows of the indicated genotype was revealed. This was expressed in the shortest duration of coagulation, the formation of better gel strength, and increased yield relative to samples obtained from milk from animals with the *CSN3 AA* genotype. The retentates concentrated by the UF method had the best coagulation ability. The trend of the positive effect of the *CSN3 BB* genotype with acid, rennet, and acid–rennet coagulation of UF-, NF-, and RO-retentate samples was revealed, evidence to the greatest extent with UF-treatment. A higher protein transition from UF-, NF-, and RO-retentates to clots obtained by both heat- and calcium-induced coagulation, and heat- and acid-induced coagulation was detected in *CSN3 AA* genotype samples, regardless of the type of pressure-driven membrane processes. Thus, the cumulative effect of such biomolecular and biotechnological factors such as *CSN3* genetic polymorphism; pressure-driven membrane processes of concentrating dairy systems; and the sensitivity of the protein cluster to the effects of various coagulation methods on the formation of the technological potential of milk, namely, its structure formation, was established. This study will improve the efficiency of milk processing and optimize the technology of dairy product production. It will also expand the criteria to evaluate its technological properties and purchase pricing systems, thus helping to stimulate the market of milk producers and processors to diversify raw materials in accordance with its basic technological properties.

## Figures and Tables

**Table 1:** Results of *CSN3* gene genotyping of black and white breed cows.

Genotype	Number of Cows, Head	Age of Cows, Calves	Frequency Occurrence Genotypes, %	Frequency of Alleles, %	
				*A*	*B*
*AA*	225	2.3 ± 0.1	63.7	77.5	22.5
*AB*	97	2.6 ± 0.1	27.5		
*BB*	31	2.1 ± 0.2	8.8		

**Table 2:** Analysis of milk characteristics estimated on genetic variant *CSN3* in the investigated population.

Genotype	Milk Yield (kg)	Fat Content (%)	Fat Yield (kg)	Protein Contnet (%)	Protein Yield (kg)
*AA*	10,359 ± 443	3.69 ± 0.15	382.3 ± 15.5	3.22 ± 0.07	333.6 ± 7.3
*AB*	10,297 ± 405	3.71 ± 0.12	382.0 ± 12.4	3.17 ± 0.13	326.4 ± 13.4
*BB*	9983 ± 381	3.88 ± 0.21	387.3 ± 21.0	3.30 ± 0.09	329.4 ± 9.0

**Table 3:** Physico-chemical composition of skimmed milk, retentates, and concentrates.

Name of Parameter	Skim Milk			UF-Retentat			NF-Retentat			RO-Retentat			VE-Concentrat		
	CSN3^AA^	CSN3^AB^	CSN3^BB^	CSN3^AA^	CSN3^AB^	CSN3^BB^	CSN3^AA^	CSN3^AB^	CSN3^BB^	CSN3^AA^	CSN3^AB^	CSN3^BB^	CSN3^AA^	CSN3^AB^	CSN3^BB^
Fat, %	0.07 ± 0.01 ^a^	0.06 ± 0.01 ^a^	0.10 ± 0.01 ^b^	0.14 ± 0.02 ^b^	0.12 ± 0.01 ^b^	0.20 ± 0.03 ^c^	0.14 ± 0.02 ^b^	0.12 ± 0.02 ^b^	0.19 ± 0.03 ^c^	0.14 ± 0.01 ^b^	0.12 ± 0.02 ^b^	0.19 ± 0.03 ^c^	0.15 ± 0.02 ^bc^	0.12 ± 0.02 ^b^	0.20 ± 0.02 ^c^
Protein, %	3.30 ± 0.12 ^a^	3.24 ± 0.09 ^a^	3.39 ± 0.10 ^a^	6.64 ± 0.12 ^b^	6.62 ± 0.10 ^b^	6.61 ± 0.09 ^b^	6.63 ± 0.10 ^b^	6.64 ± 0.12 ^b^	6.61 ± 0.10 ^b^	6.62 ± 0.11 ^b^	6.64 ± 0.14 ^b^	6.61 ± 0.09 ^b^	6.60 ± 0.09 ^b^	6.63 ± 0.13 ^b^	6.64 ± 0.11 ^b^
Casein, %	2.58 ± 0.09 ^a^	2.53 ± 0.14 ^a^	2.71 ± 0.14 ^a^	5.58 ± 0.09 ^b^	5.53 ± 0.09 ^b^	5.63 ± 0.11 ^b^	5.16 ± 0.10 ^c^	5.19 ± 0.14 ^c^	5.29 ± 0.12 ^c^	5.16 ± 0.14 ^c^	5.20 ± 0.13 ^c^	5.29 ± 0.10 ^c^	5.16 ± 0.09 ^c^	5.18 ± 0.14 ^c^	5.32 ± 0.10 ^c^
αS1-CN	1.13 ± 0.03 ^a^	1.11 ± 0.03 ^a^	1.20 ± 0.04 ^a^	2.39 ± 0.07 ^b^	2.41 ± 0.05 ^b^	2.50 ± 0.08 ^b^	2.28 ± 0.04 ^c^	2.26 ± 0.06 ^c^	2.32 ± 0.03 ^c^	2.29 ± 0.04 ^c^	2.26 ± 0.04 ^c^	2.35 ± 0.03 ^bc^	2.24 ± 0.04 ^c^	2.25 ± 0.02 ^c^	2.33 ± 0.06 ^c^
αS2-CN	0.25 ± 0.02 ^a^	0.26 ± 0.02 ^a^	0.23 ± 0.01 ^a^	0.55 ± 0.03 ^b^	0.53 ± 0.02 ^b^	0.47 ± 0.04 ^c^	0.48 ± 0.02 ^c^	0.53 ± 0.04 ^bc^	0.46 ± 0.03 ^c^	0.49 ± 0.02 ^c^	0.53 ± 0.01 ^b^	0.45 ± 0.03 ^c^	0.51 ± 0.03 ^bc^	0.54 ± 0.02 ^b^	0.46 ± 0.01 ^c^
β-CN	0.97 ± 0.03 ^a^	0.92 ± 0.02 ^a^	0.98 ± 0.02 ^a^	2.12 ± 0.02 ^b^	2.05 ± 0.03 ^b^	2.03 ± 0.03 ^b^	1.92 ± 0.05 ^c^	1.90 ± 0.02 ^c^	1.93 ± 0.03 ^c^	1.96 ± 0.05 ^c^	1.91 ± 0.04 ^c^	1.90 ± 0.02 ^c^	1.96 ± 0.05 ^c^	1.90 ± 0.03 ^c^	1.93 ± 0.02 ^c^
κ-CN	0.23 ± 0.02 ^a^	0.24 ± 0.01 ^a^	0.30 ± 0.02 ^b^	0.52 ± 0.01 ^c^	0.54 ± 0.02 ^c^	0.61 ± 0.02 ^d^	0.48 ± 0.01 ^e^	0.50 ± 0.01 ^c^	0.58 ± 0.02 ^d^	0.46 ± 0.02 ^e^	0.50 ± 0.02 ^c^	0.57 ± 0.03 ^cd^	0.45 ± 0.01 ^e^	0.49 ± 0.01 ^c^	0.60 ± 0.03 ^d^
Whey protein, %	0.72 ± 0.07 ^a^	0.71 ± 0.05 ^a^	0.68 ± 0.05 ^a^	1.06 ± 0.03 ^b^	1.09 ± 0.04 ^b^	0.98 ± 0.04 ^b^	1.47 ± 0.03 ^c^	1.45 ± 0.06 ^c^	1.32 ± 0.04 ^d^	1.46 ± 0.03 ^c^	1.44 ± 0.05 ^c^	1.32 ± 0.05 ^d^	1.44 ± 0.07 ^c^	1.45 ± 0.04 ^c^	1.32 ± 0.03 ^d^
β-LG	0.40 ± 0.03 ^a^	0.38 ± 0.03 ^a^	0.40 ± 0.04 ^a^	0.57 ± 0.03 ^b^	0.56 ± 0.02 ^b^	0.55 ± 0.03 ^b^	0.79 ± 0.04 ^c^	0.76 ± 0.05 ^c^	0.75 ± 0.03 ^c^	0.81 ± 0.05 ^c^	0.78 ± 0.04 ^c^	0.79 ± 0.04 ^c^	0.82 ± 0.04 ^c^	0.79 ± 0.02 ^c^	0.76 ± 0.03 ^c^
α-LA	0.17 ± 0.03 ^a^	0.18 ± 0.03 ^a^	0.14 ± 0.03 ^a^	0.22 ± 0.02 ^a b^	0.24 ± 0.04 ^ab^	0.18 ± 0.03 ^a^	0.32 ± 0.03 ^c^	0.34 ± 0.02 ^c^	0.26 ± 0.01 ^b^	0.33 ± 0.02 ^c^	0.38 ± 0.03 ^c^	0.27 ± 0.01 ^b^	0.33 ± 0.02 ^c^	0.36 ± 0.01 ^c^	0.29 ± 0.02 ^bc^
BSA	0.03 ± 0.002 ^a^	0.03 ± 0.002 ^a^	0.04 ± 0.003 ^b^	0.06 ± 0.002 ^c^	0.06 ± 0.002 ^c^	0.08 ± 0.002 ^d^	0.06 ± 0.001 ^c^	0.06 ± 0.002 ^c^	0.08 ± 0.002 ^d^	0.06 ± 0.002 ^c^	0.05 ± 0.001 ^e^	0.07 ± 0.003 ^f^	0.05 ± 0.001 ^e^	0.06 ± 0.003 ^c^	0.08 ± 0.003 ^d^
Lactose, %	5.01 ± 0.12 ^a^	4.96 ± 0.09 ^a^	5.10 ± 0.14 ^a^	5.26 ± 0.16 ^a^	5.31 ± 0.14 ^a^	5.27 ± 0.17 ^b^	9.66 ± 0.18 ^b^	9.84 ± 0.21 ^b^	9.65 ± 0.15 ^b^	10.15 ± 0.19 ^b^	10.18 ± 0.20 ^b^	9.95 ± 0.16 ^b^	10.01 ± 0.21 ^b^	10.17 ± 0.21 ^b^	10.00 ± 0.19 ^b^
Ash, %	0.76 ± 0.03 ^a^	0.72 ± 0.01 ^a^	0.79 ± 0.03 ^a^	0.88 ± 0.03 ^a^	0.85 ± 0.04 ^a^	0.96 ± 0.04 ^a^	1.21 ± 0.02 ^b^	1.14 ± 0.04 ^b^	1.23 ± 0.02 ^b^	1.44 ± 0.03 ^c^	1.39 ± 0.03 ^c^	1.46 ± 0.04 ^c^	1.44 ± 0.02 ^c^	1.40 ± 0.04 ^c^	1.55 ± 0.04 ^c^
Calcium, mg/100 g	119.37 ± 3.15 ^a^	122.19 ± 3.76 ^a^	131.49 ± 3.48 ^a^	186.39 ± 4.11 ^b^	191.53 ± 4.23 ^b^	203.47 ± 4.15 ^c^	201.18 ± 4.15 ^c^	205.36 ± 3.95 ^c^	219.79 ± 3.87 ^d^	233.83 ± 3.78 ^e^	239.35 ± 4.02 ^e^	251.57 ± 4.13 ^f^	235.71 ± 3.56 ^e^	248.03 ± 3.92 ^e^	252.48 ± 4.15 ^f^
Magnesium, mg/100 g	13.71 ± 0.22 ^a^	13.87 ± 0.24 ^a^	14.64 ± 0.27 ^a^	16.28 ± 0.33 ^b^	16.68 ± 0.18 ^b^	17.32 ± 0.18 ^b^	23.56 ± 0.24 ^c^	24.04 ± 0.26 ^c^	24.98 ± 0.19 ^c^	26.23 ± 0.29 ^d^	27.16 ± 0.27 ^d^	27.36 ± 0.31 ^d^	26.45 ± 0.32 ^d^	27.49 ± 0.24 ^d^	27.91 ± 0.43 ^d^
Sodium, mg/100 g	24.87 ± 0.33 ^a^	24.35 ± 0.18 ^a^	24.14 ± 0.21 ^a^	25.96 ± 0.27 ^a^	25.52 ± 0.24 ^a^	24.91 ± 0.20 ^a^	34.80 ± 0.31 ^b^	34.07 ± 0.34 ^b^	33.77 ± 0.41 ^b^	48.15 ± 0.21 ^c^	47.15 ± 0.42 ^c^	46.74 ± 0.26 ^c^	48.84 ± 0.31 ^c^	48.53 ± 0.39 ^c^	46.68 ± 0.27 ^c^
Potassium, mg/100 g	164.08 ± 2.15 ^a^	162.03 ± 1.76 ^a^	158.45 ± 1.82 ^a^	126.57 ± 3.11 ^b^	124.13 ± 1.97 ^b^	121.96 ± 2.13 ^b^	203.51 ± 3.26 ^c^	201.04 ± 3.17 ^c^	196.95 ± 2.66 ^c^	317.65 ± 3.51 ^d^	319.18 ± 3.42 ^d^	298.38 ± 3.45 ^e^	322.33 ± 4.15 ^d^	325.31 ± 4.25 ^d^	304.57 ± 4.03 ^e^
Phosphates, mg/100 g	118.42 ± 4.12 ^a^	110.35 ± 3.19 ^a^	104.00 ± 3.03 ^a^	154.43 ± 3.75 ^b^	143.90 ± 3.82 ^b^	135.62 ± 3.24 ^ab^	191.83 ± 2.99 ^c^	178.76 ± 3.18 ^c^	168.47 ± 3.42 ^bc^	222.03 ± 3.87 ^d^	208.94 ± 4.02 ^d^	195.65 ± 3.94 ^c^	233.91 ± 3.75 ^e^	222.54 ± 4.00 ^d^	201.46 ± 4.15 ^c^
Citrates, mg/100 g	173.01 ± 3.02 ^a^	151.21 ± 3.41 ^b^	122.18 ± 2.95 ^c^	208.98 ± 3.45 ^d^	194.27 ± 3.12 ^a^	147.84 ± 3.05 ^c^	257.38 ± 3.28 ^e^	220.77 ± 3.21 ^d^	179.51 ± 3.01 ^a^	337.90 ± 3.12 ^f^	301.61 ± 3.41 ^g^	232.53 ± 3.30 ^d^	341.13 ± 3.64 ^f^	306.15 ± 3.7 ^g^	235.12 ± 3.32 ^d^
Total solids, %	9.41 ± 0.17 ^a^	9.21 ± 0.18 ^a^	9.57 ± 0.14 ^a^	13.97 ± 0.16 ^b^	13.91 ± 0.15 ^b^	14.14 ± 0.19 ^b^	17.81 ± 0.14 ^c^	17.98 ± 0.22 ^c^	17.79 ± 0.19 ^c^	18.47 ± 0.20 ^c^	18.45 ± 0.20 ^c^	18.36 ± 0.17 ^c^	18.76 ± 0.21 ^c^	18.45 ± 0.20 ^c^	19.10 ± 0.20 ^c^
Titratable acidity, °T	16.5 ± 0.5 ^a^	16.4 ± 0.4 ^a^	17.0 ± 0.5 ^a^	27.0 ± 0.3 ^a^	27.5 ± 0.4 ^a^	25.6 ± 0.3 ^a^	23.5 ± 0.3 ^a^	23.4 ± 0.5 ^a^	25.0 ± 0.2 ^a^	39.1 ± 0.5 ^a^	38.5 ± 0.6 ^a^	36.8 ± 0.6 ^a^	35.2 ± 0.4 ^a^	34.9 ± 0.5 ^a^	36.1 ± 0.5 ^a^
pH	6.79 ± 0.04 ^a^	6.77 ± 0.04 ^a^	6.75 ± 0.05 ^a^	6.81 ± 0.02 ^b^	6.79 ± 0.05 ^b^	6.75 ± 0.03 ^c^	6.71 ± 0.03 ^c^	6.73 ± 0.04 ^c^	6.69 ± 0.02 ^c^	6.50 ± 0.04 ^d^	6.52 ± 0.05 ^d^	6.54 ± 0.03 ^e^	6.49 ± 0.03 ^e^	6.51 ± 0.02 ^e^	6.42 ± 0.04 ^e^

^a–g^ Means followed by different lowercase letters in rows differ significantly by ANOVA; significance level of 5%. UF-CSN3^AA-BB^; NF-CSN3^AA-BB^; RO- CSN3^AA-BB^; VE- CSN3^AA-BB^—sample names with the annotated corresponding genotype.

**Table 4:** Physico-chemical parameters of clots of skimmed milk samples and UF, NF, RO retents depending on *CSN3* polymorphism.

Name of Parameter	Enzyme Preparations							
	ME	BCP 96/04	BCP 50/50	BCP 05/95	ME	BCP 96/04	BCP 50/50	BCP 05/95
	CSN3^AA^				CSN3^BB^			
Clot yield, %	15.2 ± 0.3 ^a^	14.6 ± 0.2 ^b^	15.8 ± 0.3 ^c^	16.0 ± 0.3 ^c^	16.1 ± 0.2 ^c^	15.4 ± 0.1 ^a^	16.6 ± 0.4 ^c^	17.2 ± 0.4 ^d^
Total solids, %	22.50 ± 0.21 ^a^	23.04 ± 0.34 ^b^	21.56 ± 0.17 ^c^	21.93 ± 0.14 ^c^	23.10 ± 0.27 ^b^	23.50 ± 0.25 ^b^	22.48 ± 0.19 ^a^	22.15 ± 0.17 ^a^
Protein, %	16.19 ± 0.13 ^a^	17.71 ± 0.15 ^b^	16.16 ± 0.10 ^a^	15.84 ± 0.08 ^a^	16.85 ± 0.16 ^c^	18.07 ± 0.14 ^b^	16.45 ± 0.11 ^a^	16.07 ± 0.09 ^a^
Protein retention factor	0.746 ± 0.006 ^a^	0.784 ± 0.011 ^b^	0.774 ± 0.009 ^b^	0.768 ± 0.009 ^b^	0.800 ± 0.006 ^c^	0.821 ± 0.012 ^c^	0.806 ± 0.001 ^c^	0.815 ± 0.001 ^c^
	UF-CSN3^AA^				UF-CSN3^BB^			
Clot yield, %	27.3 ± 0.5 ^a^	26.5 ± 0.6 ^a^	27.4 ± 0.5 ^a^	28.7 ± 0.7 ^b^	28.3 ± 0.7 ^b^	27.6 ± 0.5 ^ab^	28.2 ± 0.7 ^ab^	29.6 ± 0.8 ^b^
Total solids, %	24.61 ± 0.25 ^a^	24.74 ± 0.24 ^a^	24.31 ± 0.21 ^a^	24.25 ± 0.26 ^a^	27.71 ± 0.31 ^b^	28.03 ± 0.27 ^b^	26.89 ± 0.24 ^b^	26.52 ± 0.19 ^b^
Protein, %	21.32 ± 0.16 ^a^	21.80 ± 0.18 ^a^	21.15 ± 0.16 ^a^	19.01 ± 0.14 ^b^	20.43 ± 0.17 ^c^	21.85 ± 0.20 ^a^	20.61 ± 0.15 ^c^	20.12 ± 0.14 ^c^
Protein retention factor	0.882 ± 0.001 ^a^	0.875 ± 0.006 ^a^	0.878 ± 0.002 ^a^	0.827 ± 0.006 ^b^	0.876 ± 0.016 ^a^	0.914 ± 0.011 ^c^	0.881 ± 0.015 ^a^	0.902 ± 0.017 ^ac^
	NF-CSN3^AA^				NF-CSN3^BB^			
Clot yield, %	29.6 ± 0.6 ^a^	29.0 ± 0.7 ^a^	29.4 ± 0.5 ^a^	30.9 ± 0.9 ^ab^	30.9 ± 0.7 ^ab^	30.4 ± 0.6 ^ab^	30.2 ± 0.6 ^ab^	31.6 ± 0.8 ^b^
Total solids, %	25.34 ± 0.24 ^a^	25.52 ± 0.26 ^a^	24.76 ± 0.21 ^a^	23.49 ± 0.19 ^b^	24.03 ± 0.23 ^b^	25.41 ± 0.23 ^a^	25.06 ± 0.27 ^a^	24.96 ± 0.21 ^a^
Protein, %	20.05 ± 0.16 ^a^	20.31 ± 0.18 ^a^	19.87 ± 0.16 ^a^	18.61 ± 0.14 ^b^	17.97 ± 0.17 ^b^	20.00 ± 0.19 ^a^	18.69 ± 0.15 ^b^	18.59 ± 0.17 ^b^
Protein retention factor	0.899 ± 0.008 ^a^	0.892 ± 0.012 ^a^	0.885 ± 0.005 ^a^	0.871 ± 0.015 ^a^	0.841 ± 0.018 ^b^	0.921 ± 0.012 ^c^	0.855 ± 0.010 ^b^	0.890 ± 0.016 ^a^
	RO-CSN3^AA^				RO-CSN3^BB^			
Clot yield, %	29.4 ± 0.6 ^a^	28.7 ± 0.5 ^a^	29.1 ± 0.7 ^a^	30.9 ± 0.9 ^b^	30.9 ± 0.7 ^b^	29.2 ± 0.4 ^a^	29.9 ± 0.6 ^a^	31.4 ± 0.9 ^b^
Total solids, %	28.30 ± 0.27 ^a^	28.66 ± 0.24 ^a^	27.96 ± 0.25 ^b^	27.68 ± 0.19 ^b^	27.89 ± 0.24 ^b^	28.73 ± 0.29 ^a^	27.96 ± 0.18 ^b^	27.81 ± 0.21 ^b^
Protein, %	20.04 ± 0.18 ^a^	21.11 ± 0.16 ^b^	19.86 ± 0.14 ^a^	18.48 ± 0.18 ^c^	18.50 ± 0.19 ^c^	21.07 ± 0.21 ^b^	19.12 ± 0.15 ^c^	18.81 ± 0.17 ^c^
Protein retention factor	0.893 ± 0.008 ^a^	0.918 ± 0.04 ^a^	0.876 ± 0.010 ^a^	0.865 ± 0.017 ^a^	0.866 ± 0.016 ^a^	0.932 ± 0.008 ^b^	0.866 ± 0.011 ^a^	0.895 ± 0.020 ^a^

^a–d^ Means followed by different lowercase letters in columns differ significantly by ANOVA; significance level of 5%.

**Table 5:** Physico-chemical parameters of clots of skimmed milk samples, UF, NF, RO retentates, and concentrates obtained under the action of various biochemical and chemical methods of protein destabilization (coagulation), depending on *CSN3* polymorphism.

Name of Parameter	Protein Coagulation Method							
	AC	ARC	TCC	TAC	AC	ARC	TCC	TAC
	CSN3^AA^				CSN3^BB^			
Coagulation duration, min	600 ± 5 ^aA^	480 ± 5 ^bA^	5.0 ± 0.1	10 ± 1	600 ± 5 ^aA^	480 ± 5 ^bA^	5.0 ± 0.1	10 ± 1
Clot yield, %	13.9 ± 0.4 ^a^	14.8 ± 0.5 ^a^	15.9 ± 0.7 ^b^	20.7 ± 0.5 ^c^	16.2 ± 0.3 ^b^	15.8 ± 0.4 ^b^	16.3 ± 0.4 ^b^	16.6 ± 0.5 ^b^
Total solids, %	26.60 ± 0.21 ^a^	25.11 ± 0.19 ^b^	27.53 ± 0.24 ^c^	23.60 ± 0.22 ^b^	24.55 ± 0.23 ^b^	25.84 ± 0.19 ^b^	27.52 ± 0.25 ^c^	28.31 ± 0.27 ^c^
Protein, %	20.26 ± 0.16	18.93 ± 0.14	19.97 ± 0.17	15.31 ± 0.18	17.37 ± 0.17	19.31 ± 0.15	19.44 ± 0.19	19.80 ± 0.23
	UF-CSN3^AA^				UF-CSN3^BB^			
Coagulation duration, min	690 ± 5 ^aB^	520 ± 5 ^bB^	5.0 ± 0.1	10 ± 1	670 ± 5 ^cB^	505 ± 5 ^bAB^	5.0 ± 0.1	10 ± 1
Clot yield, %	29.7 ± 0.8 ^a^	27.9 ± 0.7 ^a^	37.3 ± 1.1 ^b^	25.2 ± 0.6 ^a^	33.4 ± 0.9 ^a^	28.0 ± 0.6 ^a^	41.5 ± 1.0 ^c^	24.9 ± 0.6 ^a^
Total solids, %	25.62 ± 0.24 ^a^	25.13 ± 0.22 ^a^	23.64 ± 0.19 ^a^	31.44 ± 0.29 ^b^	23.81 ± 0.17 ^a^	25.78 ± 0.21 ^a^	18.38 ± 0.15 ^c^	29.90 ± 0.27 ^b^
Protein, %	20.24 ± 0.14	20.95 ± 0.16	16.97 ± 0.13	25.55 ± 0.21	18.16 ± 0.14	21.32 ± 0.18	14.86 ± 0.11	25.40 ± 0.20
	NF-CSN3^AA^				NF-CSN3^BB^			
Coagulation duration, min	700 ± 5 ^aB^	540 ± 5 ^bC^	5.0 ± 0.1	10 ± 1	690 ± 5 ^aC^	525 ± 5 ^bB^	5.0 ± 0.1	10 ± 1
Clot yield, %	31.1 ± 0.9 ^a^	29.6 ± 0.7 ^a^	38.3 ± 1.0 ^b^	24.9 ± 0.5 ^c^	31.3 ± 0.9 ^a^	29.7 ± 0.7 ^a^	41.3 ± 1.1 ^b^	26.5 ± 0.8 ^c^
Total solids, %	22.68 ± 0.19 ^a^	23.16 ± 0.24 ^a^	25.46 ± 0.23 ^a^	35.43 ± 0.27 ^b^	23.24 ± 0.21 ^a^	23.61 ± 0.23 ^a^	23.25 ± 0.19 ^a^	36.03 ± 0.32 ^b^
Protein, %	18.44 ± 0.14	19.23 ± 0.19	16.42 ± 0.18	25.91 ± 0.24	18.57 ± 0.16	19.52 ± 0.15	15.03 ± 0.15	23.93 ± 0.27
	RO-CSN3^AA^				RO-CSN3^BB^			
Coagulation duration, min	690 ± 5 ^aB^	540 ± 5 ^bC^	5.0 ± 0.1	10 ± 1	670 ± 5 ^cB^	525 ± 5 ^bB^	5.0 ± 0.1	10 ± 1
Clot yield, %	30.5 ± 0.7 ^a^	29.1 ± 0.5 ^a^	38.0 ± 1.1 ^b^	24.8 ± 0.4 ^c^	30.7 ± 0.9 ^a^	29.2 ± 0.6 ^a^	40.9 ± 1.1 ^b^	26.3 ± 0.8 ^c^
Total solids, %	26.17 ± 0.24 ^a^	27.26 ± 0.27 ^a^	25.71 ± 0.23 ^a^	35.55 ± 0.31 ^b^	26.06 ± 0.24 ^a^	27.59 ± 0.28 ^a^	23.43 ± 0.19 ^c^	35.74 ± 0.30 ^b^
Protein, %	19.31 ± 0.19	20.11 ± 0.22	16.59 ± 0.18	26.03 ± 0.26	19.24 ± 0.20	20.27 ± 0.22	15.28 ± 0.14	24.15 ± 0.25
	VE-CSN3^AA^				VE-CSN3^BB^			
Coagulation duration, min	650 ± 5 ^aC^	550 ± 5 ^bC^	5.0 ± 0.1	10 ± 1	645 ± 5 ^aB^	550 ± 5 ^bC^	5.0 ± 0.1	10 ± 1
Clot yield, %	42.7 ± 1.2 ^a^	41.7 ± 1.0 ^a^	29.7 ± 0.7 ^b^	27.0 ± 0.5 ^b^	43.1 ± 1.0 ^a^	41.9 ± 0.9 ^a^	31.1 ± 0.7 ^b^	27.1 ± 0.5 ^b^
Total solids, %	21.30 ± 0.20 ^a^	20.80 ± 0.18 ^a^	31.03 ± 0.27 ^b^	31.18 ± 0.30 ^b^	21.21 ± 0.19 ^a^	20.95 ± 0.18 ^a^	28.74 ± 0.24 ^c^	31.43 ± 0.28 ^b^
Protein, %	13.42 ± 0.17	13.60 ± 0.14	20.24 ± 0.22	22.89 ± 0.24	13.35 ± 0.12	13.72 ± 0.13	19.03 ± 0.18	22.31 ± 0.20

^a–c^ Means followed by different lowercase letters in rows differ significantly by ANOVA; significance level of 5%. ^A–C^ Means followed by different uppercase letters in columns differ significantly by ANOVA; significance level of 5%.

## Data Availability

Data is contained within the article.
